# Betwixt and between: Role conflict, role ambiguity and role definition in project-based dual-leadership structures

**DOI:** 10.1177/0018726717692852

**Published:** 2017-04-28

**Authors:** Joris J Ebbers, Nachoem M Wijnberg

**Affiliations:** University of Amsterdam, the Netherlands, j.j.ebbers@uva.nl; University of Amsterdam, the Netherlands and University of Johannesburg, South Africa, n.m.wijnberg@uva.nl

**Keywords:** creative industries, dual leadership, film industry, project-based organization, role crafting

## Abstract

Project-based organizations in the film industry usually have a dual-leadership structure, based on a division of tasks between the dual leaders – the director and the producer – in which the former is predominantly responsible for the artistic and the latter for the commercial aspects of the film. These organizations also have a role hierarchically below and between the dual leaders: the 1st assistant director. This organizational constellation is likely to lead to role conflict and role ambiguity experienced by the person occupying that particular role. Although prior studies found negative effects of role conflict and role ambiguity, this study shows they can also have beneficial effects because they create space for defining the role expansively that, in turn, can be facilitated by the dual leaders defining their own roles more narrowly. In a more general sense, this study also shows the usefulness of analyzing the antecedents and consequences of roles, role definition, and role crafting in connection to the behavior of occupants of adjacent roles.

## Introduction

Organizations with a dual-leadership structure are those in which two leaders share the highest position in the hierarchy (Reid and Karambayya, 2009). These dual-leadership structures belong to the broader class of plural leadership forms that are characterized by collective leadership (Denis et al., 2012). One general problem of distributing leadership roles over multiple individuals is that it creates the risk of conflict between the leaders themselves (Reid and Karambayya, 2009), while also increasing the risk that other members of the organization get involved in these leadership conflicts.

With regard to intra-organizational conflicts, prior research often distinguishes between task conflict and relationship conflict. Task conflict means that there is disagreement among members of the organization about the content of the task being performed by one or more of them. Relationship conflict, on the other hand, denotes that there are interpersonal incompatibilities between the members, expressed by tensions, hostility or annoyance (Jehn, 1995). Although a certain degree of task conflict can have positive outcomes, because discussion and debate can lead to creativity and better decision-making, relationship conflict is generally considered to be detrimental to performance and satisfaction (Jehn, 1997).

Task conflict is related to role boundaries between organizational members, which can be especially acute in plural leadership structures because task conflict between plural leaders, as well as between them and other members of the organization, can lead to role stress. Role stress can be divided into role conflict and role ambiguity. Role conflict arises when individuals are faced with inconsistent or incompatible demands (Biddle, 1986; House and Rizzo, 1972; Kahn et al., 1964; Rizzo et al., 1970), whereas role ambiguity refers to uncertainty about which tasks and responsibilities are part of the role (Biddle, 1986). Prior studies suggest that organizations should minimize role conflict and role ambiguity because of its negative effects, such as dissatisfaction, anxiety, lower commitment and lower performance (House and Rizzo, 1972; Jackson and Schuler, 1985; Tubre and Collins, 2000).

In this study, we explore how role conflict and role ambiguity can also have positive effects, precisely because they force individuals to define their role. Role definition denotes how individuals perceive their role and is often used to distinguish between in-role behavior and extra-role behavior (Morrison, 1994; Sluss et al., 2011; Tepper et al., 2001). When organizational members define their roles this, in turn, can lead to others in adjacent roles subsequently redefining their roles, which can lead to positive outcomes for the organization as a whole. Besides being a process of drawing conclusions from the available information about what one is expected to do, role definition can also be understood as a first phase in role crafting (Sluss et al., 2011).

In this article, we study project-based organizations (PBOs) with a dual-leadership structure and focus on the definition of the role hierarchically below and between the dual leaders.

In PBOs, the negative effects of role conflict and ambiguity can cause even greater problems, because many such organizations, especially in the service and creative industries, benefit from the strong emotional commitments from their members (Lindgren et al., 2014; Rowlands and Handy, 2012), and role conflict and role ambiguity can interfere with the positive emotions of the individuals involved. Precisely because of the temporary nature of PBOs, roles in such organizations are often fairly standardized. This allows the professionals involved in the project to more easily work with others they have never collaborated with before (Baker and Faulkner, 1991; Bechky, 2006; Rowlands and Handy, 2012). At the same time, one could expect employees in PBOs to expansively define their roles to stand out from the rest and improve their chances of getting hired for attractive projects or more responsible positions in the future (Ebbers and Wijnberg, 2009b).

Although dual leaders can share the same responsibilities and objectives, a dual-leadership structure usually means that the two leaders have different responsibilities and objectives (Denis et al., 2000; Reid and Karambayya, 2009). The literature on dual leadership mostly focuses on the dual leaders and the relation between them (Reid and Karambayya, 2009). Instead, this study does not just look at the dual leaders in relation to each other, but especially in relation to the persons immediately below them. When dual leaders have a clear division of responsibilities and objectives, individuals occupying a role between and hierarchically below the dual leaders are likely to experience role conflict and role ambiguity. However, as we will argue, these circumstances also enable this person to expansively define their role, which can create benefits for the organization as a whole.

This study therefore addresses two main questions. First, how do individuals occupying a role hierarchically below and between the dual leaders deal with the role ambiguity and role conflict inherent to their role? Second, how can the dual leaders affect the opportunities these individuals have to define their roles expansively?

The empirical setting of our qualitative study is the film industry. Films are often produced in PBOs with a dual-leadership structure consisting of the director and producer, who are predominantly responsible for artistic and commercial matters, respectively (Baker and Faulkner, 1991; Rowlands and Handy, 2012; Svejenova et al., 2010). Because of its project-based nature, most roles in the film industry are highly standardized and clearly defined to ensure smooth collaboration between professionals that often do not have a collaboration history (Bechky, 2006). However, this is much less the case for the particular role on which we focus in this article: the 1st assistant director (1st AD). The 1st AD occupies a position hierarchically below the producer and director, and has to report to both. Because this role is inherently characterized by role conflict and role ambiguity, the 1st AD needs to define her or his role in each different project. We therefore conducted interviews with 1st ADs. These data were supplemented with those from interviews with producers and directors in prior research projects (Ebbers and Wijnberg, 2009a, 2009b) that raised our interest in the role of the 1st AD, especially in relation to the dual leaders.

## Theoretical framework

### Roles, role definition and role crafting

An organizational role is a position within an organizational structure that comes with a specified set of tasks or responsibilities (House and Rizzo, 1972). Alternatively, roles have been described as socially constructed units of what is appropriate and expected of a person in a particular position in an organization or team (Ilgen and Hollenbeck, 1990). A person who occupies a particular role is expected to perform the associated set of tasks and make decisions that are fitting to that role (Rogers and Molnar, 1976). In many cases, roles change over time and are (re-)designed top down by managers or employers to deal with changing circumstances. Yet the exact role boundaries are often ill-defined by managers or employers (Kahn et al., 1964), as a result of which employees, at least to some extent, need to define their own role.

The literature on role definition is concerned with how individuals perceive the boundaries of their role within their organization, and is often used to distinguish in-role from extra-role behavior, especially in relation to organizational citizenship behavior (Morrison, 1994; Sluss et al., 2011; Tepper et al., 2001). Employees with the same job can differ in how they define the boundaries of their role. The more activities they perceive to be in-role instead of extra-role, the larger their perceived job breadth, and the more broadly they define their role (Morrison, 1994). As a result, role definition can lead to employees either reducing or expanding their activities. Role definition can sometimes be considered as the first phase of role crafting, which can be defined as the ‘establishment and subsequent change of roles within organizations’ (Sluss et al., 2011: 515). Wrzesniewski and Dutton (2001) define job – not role – crafting as ‘the physical and cognitive changes individuals make in the task or relational boundaries of their work’.

In this study, we focus on role definition in relation to internal boundary spanning, in which individuals forge links between different divisions or departments in their organization (Perrone et al., 2003; Rosenkopf and Nerkar, 2001). Because most roles are inevitably linked to other roles within the organization, the extent to which a particular role can be expansively defined (or crafted) depends on the particular role relationships that are associated with it (Sluss and Ashforth, 2007). Because of this interdependency, organizational members performing certain roles have expectations about those of others. Uncertainty with respect to one’s role and its boundaries can lead to role conflict and role ambiguity.

First, role conflict arises when employees are faced with inconsistent, or even incompatible, demands as to how they should behave to properly fulfill their role (Biddle, 1986; House and Rizzo, 1972; Kahn et al., 1964; Rizzo et al., 1970). Second, role ambiguity can arise when employees do not have a clear idea of the boundaries of their role, or which tasks and responsibilities are part of it (Biddle, 1986; Kahn et al., 1964). Individuals often experience both role conflict and role ambiguity at the same time because incompatible expectations associated with role conflict can interact with uncertainty about the precise content of the role (Morris et al., 1979). Moreover, an increase in role conflict can cause an increase in role ambiguity and vice versa (Rogers et al., 1994).

Role conflict and role ambiguity are often linked to undesirable outcomes for both its individual members and the organization at large. They can lead to dissatisfaction with the role, a distorted reality (Rizzo et al., 1970), decreased satisfaction, decreased organizational effectiveness (House and Rizzo, 1972), anxiety, lower commitment and lower performance (Jackson and Schuler, 1985; Tubre and Collins, 2000). It is possible to minimize role conflict and role ambiguity by designing unitary rigidly structured organizations in which all roles are explicitly described and all individuals have only one superior. However, in many contexts such organizational structures can be less suitable, and plural or dual-leadership structures, with multiple lines of authority, can seem preferable. As we will argue below, even though such structures will increase the likelihood of role conflict and role ambiguity, affected individuals can at the same time benefit from the resulting opportunities for expansive role definition, which can positively affect the organization as a whole.

### Dual leadership and role definition

When organizations are pluralistic in the sense of having multiple core objectives it can make sense to distribute these core objectives among the members of the organization by compartmentalizing the organization. Most organizations therefore tend to be divided into smaller subunits, either along the lines of product categories (for example, a firm with divisions for telecommunication networks, smartphones and cloud computing), or along functional lines (for example, by having dedicated departments for research and development, production and marketing). Although creating smaller subunits within larger organizations may lead to efficiencies, they can also create challenges in the form of weaker horizontal information exchange, and conflicts of interests between product divisions or functional departments, which, in turn, can negatively affect employee behavior and organizational performance (Galbraith, 1971; Gupta et al., 1986; Leenders and Wierenga, 2008).

The foundational idea of the matrix organization was to overcome this problem by structuring the organization along multiple lines of authority, so that each employee would have an additional manager, besides their functional manager, to report to (Ford and Randolph, 1992; Galbraith, 1971; Jones and Deckro, 1993). In matrix organizations, this additional line of authority is typically a business, program, product or project manager (Joyce, 1986). Although the matrix structure can improve cohesion among and information exchange across departments and divisions, it can also create role conflict and role ambiguity at the individual level (Knight, 1976; Sy and D’Annunzio, 2005). Although balanced matrix organizations, in which neither the functional nor the project manager is dominant, have the strongest advantages in terms of flexibility and improved information exchange, they are also the most likely to suffer from power struggles (Larson and Gobeli, 1987).

An alternative way to manage an organization is to make the multiplicity of objectives the core organizing principle and have a plural leadership structure in which the leadership roles are linked to the main organizational objectives.

The dual-leadership structure is the simplest form of pooled leadership at the top (Denis et al., 2012), with a top management team consisting of two hierarchically equivalent executives, each of which is responsible for one core objective and associated tasks (Denis et al., 2000; Reid and Karambayya, 2009). Dual-leadership structures can be found in a variety of settings, including education (Court, 2004; Fjellvaer, 2010) and healthcare (Denis et al., 2000; Fjellvaer, 2010). Yet they are especially prevalent in creative or cultural industries such as theater (Reid and Karambayya, 2009) and film (Svejenova et al., 2010). Although dual-leadership structures can attenuate power struggles, role conflict and role ambiguity at lower hierarchical levels, they also risk creating conflicts at the executive level of the dual leaders, which, in turn, can deepen conflicts or tensions in the organization as a whole.

Conflicts between dual leaders can cause major problems, especially when their objectives and responsibilities are diametrically opposed. One way of addressing this problem is to have an individual occupying a role in between the leaders, precisely to mitigate conflicts between the two. Ideally, this should be a person with informal power or influence based on expertise and ability to persuade (Galbraith, 1974: 34) or act as honest brokers (McNulty and Stewart, 2015). These persons may be especially successful if they are ‘crisscrossing actors’ who share attributes with members of each subgroup (Mäs et al., 2013). Reid and Karambayya (2009) therefore stress the importance of mediation between the two leaders. Instead of an external or ad hoc mediator, an individual structurally occupying a role between and hierarchically below the dual leaders might enable them to provide more effective leadership.

At the same time, precisely because of their position below and hierarchically between the dual leaders, these individuals may suffer from role conflict and role ambiguity. More specifically, role ambiguity can result from ambiguity with respect to occupational jurisdiction, in the sense of which occupational group has the knowledge, authority or legitimacy to control the execution of a particular range of tasks (Bechky, 2003), or who has the right to make decisions about the content, objectives and outcomes of particular tasks when there are multiple competing claims of expertise (Lingo and O’Mahony, 2010).

Even though role conflict and role ambiguity can lead to stress and other negative effects at the individual level, at the same time they can create more freedom in defining one’s role. More specifically, role ambiguity and conflict could create space for individuals to define their roles expansively. However, such expansive role definition is also likely to result in individuals partially taking over the roles of other members of the organization. For this to work well, it is therefore imperative that organizational members, whose role is being entered, have a flexible role orientation (Parker et al., 1997).

In short, because of the theoretical arguments given here, we expect role ambiguity and role conflict to be strongly present in respect to the role of the person in between and right below the dual leaders of a PBO. We expect that the effects of ambiguity and conflict are not all negative, because they could also facilitate expansive role definition. Finally, we expect the extent to which this will work out well to depend on the behavior of the dual leaders and especially the extent to which they are willing to define their roles more narrowly.

## Empirical setting: The project-based film industry

The empirical setting of our study is the film industry. The film industry can be considered an extreme case, making it a particularly illuminating context to study the phenomena of interest (Eisenhardt, 1989, Siggelkow, 2007). Prior studies show that the tension between artistic and commercial objectives is a major determinant of how organizations in cultural industries, such as the film industry, behave (eg. Caves, 2000; Cohendet and Simon, 2007; Holbrook and Addis, 2008). In many organizations in cultural industries there are leadership structures that reflect this dichotomy. In theaters, for example, one often finds a dual-leadership structure with two top executives being responsible for either the artistic or the commercial aspects of the organization (Bhansing et al., 2012). In the context of theaters, prior research on dual leadership focuses predominantly on conflicts directly between the dual leaders, with lower-level organizational members reporting to either the artistic or the commercial leader, and conflicts between dual leaders being mediated mostly by the board of directors (Reid and Karambayya, 2009).

Films are produced in temporary PBOs that dissolve once the project for which they were specifically set up is completed (DeFillippi and Arthur, 1998; Jones, 1996). In the film industry, the dichotomy between art and commerce is clearly identifiable, with the director being mainly responsible for the artistic aspects or look and feel of the film, and the producer for the business aspects that include financing and organizing (Squire, 2004). The producer and director, forming the dual leadership, can be seen as the personifications of the poles of art and commerce (Delmestri et al., 2005), because the director’s artistic goal of wanting to build a reputation based on artistic performance can potentially be hindered by the producer’s goal of keeping within budget and making a profit (Svejenova et al., 2010). However, contrary to some cultural industries such as theater, where the dual leaders are appointed by a board of directors (Reid and Karambayya, 2009), in the film industry dual leaders team up on their own initiative, and mostly for the duration of a single project. As a result, relationship conflict between dual leaders is less likely (Jehn, 1995).

In this article, we focus on the role of the 1st assistant director (1st AD). The 1st AD is positioned hierarchically below and between the producer and director and accountable to both dual leaders. In most cases, the 1st AD is involved in the pre-production phase. In this phase, problems that may arise during the shoot are signaled and tackled, and an efficient planning for the shoot is made. In the production phase, when the film is being shot, the 1st AD is responsible for executing the planning, coordinating between the different departments, and in charge of the overall film set. We specifically focus on the organizational dynamics on the set during the shoot because at this stage the tensions between art and commerce, director and producer, and time and money, come to a climax. Throughout the shoot the 1st AD is constantly communicating the intentions of the director to the rest of the cast and crew, while at the same time providing regular, often daily, progress reports to the production office. However, some attention will also be paid to the pre-production phase, when it is important to understand the dynamics during the film shoot.

**Figure 1.**
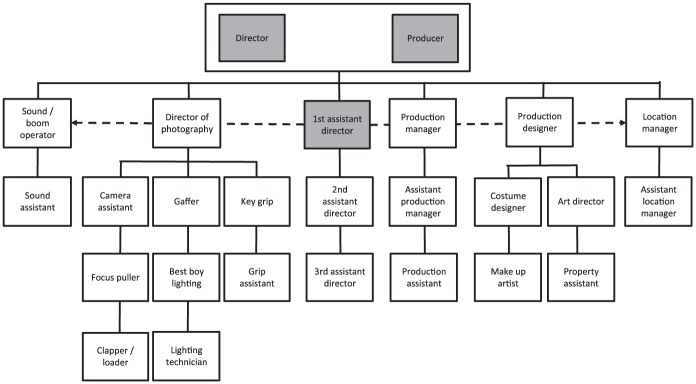
Key roles and reporting lines on a prototypical film set.

We collected data in the Dutch film industry. Although the Dutch film industry might differ from those of other countries such as the US, for example with respect to scale and unionization, the organization of the film set is very similar. Figure 1 shows the organizational structure of a prototypical film set in the Netherlands, highlighting key roles and reporting lines. This organizational structure is very similar to those that can be found in the US film industry (see Bechky, 2006). Because the focus in our article is on the producer, director and, especially, 1st AD, these roles are highlighted. The horizontal dashed lines originating from the 1st AD indicate the coordination task of the 1st AD with the heads of the other departments. As one can see, the role of the 1st AD is likely to generate role conflict and role ambiguity as a result of the potentially conflicting interests of the producer and director.

## Data and method

The present study builds on earlier projects for which we performed 24 interviews with film producers between February and June 2007, and 14 film directors between February and September 2008. This project resulted in two articles (Ebbers and Wijnberg, 2009a, 2009b). During these interviews, both directors and producers pointed out the important role of the 1st AD in managing film shoots. At the same time, it appeared that the role of 1st AD was possibly also the most stressful one, precisely because of the difficulty of balancing the interests of the dual leaders. Because we were intrigued by this role in dual-leadership structures, between April and July 2011 we conducted 14 additional semi-structured interviews with 1st ADs. Although these 14 interviews form the core data of this article, our findings have been supplemented with quotes from our earlier interviews, especially when they were concerned with the role of the 1st AD.

Our qualitative approach using semi-structured interviews to find industry-wide patterns is similar to earlier studies about project teams in creative industries (Lindgren et al., 2014; Rowlands and Handy, 2012). The fine-grained interview data about film professionals’ subjective experiences in the film industry (Rowlands and Handy, 2012) are especially appropriate for understanding organizational processes (Langley, 1999). In designing the interview protocol the use of academic terminology was limited to avoid imposing preconceived ideas on the respondents. The interviews lasted on average about 90 minutes, were tape-recorded, typed out verbatim, and sent to the respondents for their approval. We approached respondents through their professional organizations: Film Producers Netherlands, the Dutch Directors Guild and the Assistant Directors Club. To further improve the response rate, and to reduce the negative impact of socially desirable answers, informants were granted anonymity (Kumar et al., 1993).

A selection of the key questions that we used in our interviews with 1st ADs can be found in Appendix 1. On average, 1st ADs in our sample have 21.5 years of industry experience, 86% is male, 43% had previous work experience in both the production and directing departments, 43% in the production but not the directing department, 7% in the directing but not the production department, and 7% in neither of these two departments. The 14 1st ADs we interviewed correspond to 29% of the population of 1st ADs, involved in 45% of the film productions that were released in the three years before the interviews. In addition, our project confirmed that in non-probabilistic purposive samples, such as ours, data saturation occurs around 12 interviews (Guest et al., 2006). We further support our findings by using quotes from the earlier interviews with producers and directors.

In the first phase of the analysis, the first author coded quotes by producers and directors about dual leadership and the role of the 1st AD. Next, the first author coded quotes by 1st ADs that were concerned with the concepts of dual leadership, dual reporting lines, role conflict and role ambiguity. After discussion of initial findings with other scholars, attention was drawn to the concept of role crafting, and role definition as the first stage of role crafting. Next, the first author went through all the transcripts again to code quotes about role crafting or role definition. In the second phase, the second author went through the transcripts to double check the coding, and to see if relevant quotes were missing. In the third phase, an independent scholar in the field of organizational behavior checked the coding and asked critical questions by comparing the quotes with the operationalization of the concepts (Corley and Gioia, 2004). Although we initially used the concept of role crafting to code the data, at a later stage we used the label role definition, which better fitted the data because in PBOs roles are inherently temporary. One cannot observe the role after it has been crafted, because the role as such ceases to exist once the PBO is disbanded. In the last phase, we contacted a 1st AD to critically read our study. Although this person had some minor remarks and suggestions, which we addressed, she mostly confirmed our findings.

Table 1 provides a summary of the main findings. The first column highlights the three core theoretical concepts of this study: role conflict, role ambiguity and role definition. The second column highlights which 1st ADs made statements in the interviews that refer to these concepts at least once. We used a unique code (A to N) for each individual 1st AD. This enabled us to link quotes to specific respondents, while at the same time ensuring that they remain anonymous. The total number of 1st ADs referring to these three concepts is as follows: role conflict (8), role ambiguity (11) and role definition (13). The third column provides some illustrative quotes for each of the three concepts. In the next section, we will elaborate on these findings in more detail.

**Table 1.** Summary of findings and illustrative quotes.

Concept	1st AD mentions	Illustrative quotes
Role conflict	ID codes: A/B/C/D/E F/H/I/J/K/M	‘You always have two faces. You are constantly compromising. You are never able to fully support your director because you know you are there to protect the interests of the producer as well.’ (1st AD A) ‘It is a political game in which you must navigate between the wishes of the director and the opportunities that are provided by the producer. You are both the glue and the bumper between both superpowers. In some cases, one has to play the role of devil’s advocate.’ (1st AD D) ‘A director can say to you, “it is so awful that the producer is doing that”. Or the other way around a producer can say, “hey, listen, we need to see how we can navigate the director a bit in that direction because he has an enormous set of demands that we cannot meet, especially not within the limits of the budget and time. So can you do something?” That is how they use you.’ (1st AD B)
Role ambiguity	ID codes: B/C/E/F/H K/M/N	‘Sometimes I think that producers also don’t really know what to do with us. For instance, sometimes when you speak up you have the feeling that you are a pain in the neck, even though you are merely telling them that things are not going well. Then they say, “[anonymous], you are rebelling”. [I say] “I am not rebelling. I have a problem. You are the line producer, help!”’ (1st AD M) ‘You don’t know what to do and what is expected of you. You assume that there is a good working relationship between the director, the Director of Photography (DOP) and the producer and that you only need to guide them. If that is not the case, you need to be very strong and confident. In the first year I cried so often, I was at a loss. You are going through hell and at some point you figure it out.’ (1st AD F) ‘So, if I say things like, “we are moving on so we will not do that shot mister director” [I find it important] that I receive support. If the producer says [to the director], “do it anyway”, while I have been saying that it is not OK, I get angry.’ (1st AD E)
Role definition	ID codes: A/B/C/D/E/F/G I/J/K/L/M/N	‘I am not a crew member. I do not belong to the production team and I do not belong to the directing team. I am like an isolated island that needs to hold everyone together, and have everyone make compromises, in order to ultimately obtain the best possible result.’ (1st AD M) ‘It also depends on how individuals [directors and producers] enact their role and whether there is space left for me. It is possible that they are both very large and there is no way I can come between them.’ (1st AD D) ‘I hardly ever discuss things with the three of us together. I either talk to the director or the producer. That’s why I can cheat a bit: say one thing to the one person and something else to the other … For example, I give the producer advice on which arguments he can use against the director to tell him that it is possible to do it [the take] in less time.’ (1st AD G)

## Findings

### Dual leadership and the role of the 1st assistant director

Because producers and directors voluntarily team up for film projects instead of being hired, they can, at least to some extent, avoid relationship conflict. Task conflict, however, is very likely to arise. This mostly boils down to directors wanting more resources, especially in the form of hiring particular team members, or asking for extra time during the shoot, more than the producers, and the budget they control, allow. This is acknowledged by the following producer: ‘It is normal for a director to ask for 24 shooting days, while there is only room for 22 in the budget. That is fine, as long as you come to an agreement’ (Producer X). Task conflict is easier to resolve when dual leaders have a collaboration history. When directors have a long-term relationship with a producer, they are less likely to ‘stir up the crew against the producer’ (Director H). This is further confirmed by the following quotes by a producer and a director:

A director who has the long-term relationship with a producer in mind will have more eye for the balance between artistic feasibility, financial feasibility and practical feasibility from a production perspective. (Producer X)

Discussions are more to the point: What are we going to do and how are we going to do it within the boundaries of time and the financial resources that are available? … It is more about brainstorming and discussion rather than conflict. (Director N)

Task conflict between the dual leaders, especially during the shooting, can lead to a genuine threat of the organization splitting in two along the artistic–commercial divide. One of the producers mentioned the ever-present risk that a director rallies the creative team members against him or her, which can lead to a situation of ‘us [the film crew] against the production [department], or us against the people who always say: “we don’t have money for that” … But, just as well, I do not want to judge a director in terms of “but you always think the sky is the limit”’ (Producer X). Directors acknowledge this tension and point to the need to avoid an escalation of dual-leadership conflicts that might split the organization in two: ‘It is all about looking for a combination that works, just like in a marriage. Either you have your way, you agree with the other, or else you make a compromise’ (Director A).

The 1st AD is accountable to both the director and producer, and as such plays an important role in balancing the interests of the dual leaders. On the one hand, they need ‘to ensure that a situation is created in which he [the director] can optimally focus on being creative’ (1st AD E). Because there are many factors that can potentially influence the shoot, 1st ADs need to make contingency plans and make sure directors feel relaxed and confident ‘even when all hell breaks loose’ (1st AD C). On the other hand, 1st ADs have a responsibility towards the producer to finish on time and within budget. Producers therefore want to receive daily updates about the progression of the shoot and the causes of potential delays. According to the following 1st AD: ‘You are supposed to keep an eye on his [the producer’s] wallet; you are not allowed to waste any money on set’ (1st AD N). The following producer acknowledges this important role of 1st ADs: ‘They are key individuals to me. They exert all control during production’ (Producer S).

Even though the role of 1st AD is a specialization in its own right, in the Netherlands there is no formal educational degree. Some 1st ADs have prior experience in the (assistant) directing department, whereas the majority of 1st ADs in our sample have prior experience in the production department. The degree to which 1st ADs have a background in either or both the directing or production departments can affect the credibility of the 1st AD, not just in relation to the dual leaders but also the rest of the PBO:

It was a benefit that I didn’t have a production background, like many of my colleagues who are from the money [production] side … That gave me a lot more credit with the crew. Production people do not have much credit because they know little about implementation, they are not there on the set, they don’t have the practical experience and know how (1st AD H).

### The 1st assistant director and role conflict

Most 1st ADs indicate that they suffer from role conflict because it is not clear to what extent they belong to either the production or the directing department. According to one of the directors: ‘It is always the question whether the 1st AD belongs to the production department or the directing department’ (Director F). Consequently, most 1st ADs mention that they report, and are accountable, to both the producer and the director. More specifically, many say they suffer from role conflict because they are *equally* accountable to both dual leaders, which is reflected in the following quote by one of the 1st ADs: ‘My responsibilities are, by definition, difficult to reconcile because my loyalty is, and should be, 50-50’ (1st AD D). As a result, 1st ADs constantly need to balance the interests of the dual leaders. The dual reporting lines of the 1st AD towards the producer and director are also nicely illustrated by the following quote:

Technically [I am accountable] to the producer … but I am the assistant of the director, his most important assistant. In that sense, I am accountable to him as well … you are constantly thinking both practically and creatively how to save money on the one hand, and how to solve creative challenges on the other. (1st AD A)

Precisely because of their important balancing role, and the fact that a producer’s preference for a specific 1st AD can be at odds with that of the director’s, the selection of a particular 1st AD tends to be a joint 50/50 decision by the dual leaders. Producers, on the one hand, prefer 1st ADs who can complete the shoot on time and within budget. Because producers are hardly present on the film set, they try to influence the shoot via the 1st AD. In the words of one of the directors: ‘He [the 1st AD] is their bridge to the film set. Via the 1st AD he [the producer] can try to influence the pace. He does not want to become side-lined because the director and the 1st AD get too close’ (Director H). Producers may therefore refuse a particular 1st AD, when they believe he or she might neglect their interests. This is supported by the following quote by one of the 1st ADs: ‘A director wanted me for a project but production did not allow this because they thought I had chosen the side of the crew too often’ (1st AD C).

Directors, on the other hand, prefer 1st ADs who can help them achieve their artistic vision. In the words of one of the 1st ADs: ‘I think the director likes it when you have some expertise in dramaturgy that allows you to think along, which means you understand why a director makes certain choices’ (1st AD B). Directors may actually perceive it as a positive signal when a producer has a negative opinion of a particular 1st AD, and view it as an indicator that this particular 1st AD might be more sympathetic towards directors than they are to producers. This is illustrated by the following quote by one of the directors: ‘There are producers who explicitly say that they do not want to work with a specific 1st AD, but for me that is actually a reason to do precisely that’ (Director N).

During the shoot, most 1st ADs point out that they have been confronted with conflicting demands by producers and directors on many occasions. On the one hand, 1st ADs have to meet the demands of the producer by completing the shoot within the agreed upon time and budget. On the other hand, 1st ADs have to facilitate the director in realizing his or her artistic vision: ‘You have to make sure that you warrant your middle position, so that you are able to bow to the director one time and to the producer the other’ (1st AD F). When situations arise in which there is a clash between the interests of the dual leaders, it is often up to the 1st AD to come up with a solution to solve the opposing demands of the producer and director. This usually entails deleting or shortening scenes, or shooting scenes in a simpler and more economical way. This is also acknowledged by the following director: ‘He [1st AD] doesn’t just shout ‘action’, or ask how much more time you need. He should also think along with respect to the content, and must act as a bridge between production, direction, and the crew’ (Director M).

Because they are accountable to both the director and the producer, 1st ADs need to carefully balance their position between art and commerce. This means they need to ensure that they do not side too much with either the director or producer. On the one hand, 1st ADs mention that role conflict, resulting from having dual reporting lines, negatively affects their performance. This is illustrated by the following quote: ‘It may happen that a discussion takes place and someone blames you for something or heaps abuse on you, as a result of which you are dreadfully cut up by it’ (1st AD J). On the other hand, respondents emphasize that there are also positive effects of role conflict on their performance. This is illustrated by the following quote: ‘It ensures that you remain sharp, to constantly deal with new challenges, and constantly consider creative solutions, which eventually benefit the quality of the project’ (1st AD A).

### The 1st assistant director and role ambiguity

Role conflict is often accompanied by role ambiguity. Indeed, half of the 1st AD respondents mention that they experience role ambiguity. This means that the boundaries of the 1st AD role in relation to those of the dual leaders need to be (re-)discovered in each new film project. It is important to note that role conflict and role ambiguity are not always clearly separable in our empirical study. Yet this is not surprising because incompatible expectations associated with role conflict are known to interact with uncertainty about the precise content of the role (Morris et al., 1979). Moreover, an increase in role conflict can also lead to an increase in role ambiguity, and vice versa (Rogers et al., 1994).

Although in certain projects the boundaries of the 1st AD role are relatively clear from the start, in others the 1st AD needs to figure it out along the way. This is illustrated by the following quote: ‘Some producers are very involved. Other producers say: “just figure it out”. They would tell the 1st AD: “you know how much time you have for the shoot, so you figure out how to make it happen”’ (1st AD B). The degree of role ambiguity is strongly related to the fact that there are no explicit, and easy to measure, criteria to evaluate the performance of 1st ADs. Even though their overall job is to make sure that the shoot is finished on time and within budget, there are no clear guidelines as to how, or through which particular actions, to achieve this. When asked about the negative effects of not having explicit criteria that are, or could be, used to evaluate their performance, one of the 1st ADs responds:

There aren’t, because that’s just how it works. This [having explicit evaluation criteria] would mean you have to make explicit what is expected of you before you actually start. In that case, you would need to have a checklist that you could fill out at the end of the shoot, but that’s not how it works. (1st AD B)

This is further aggravated by the project-based nature of the film industry, where even an informal practice of evaluating both the project as a whole, as well as the performance of its individual members, is almost non-existent. This is illustrated by the following quote: ‘It has to do with the project-based nature of the work, the high amount of time-pressure and the fact that everybody is gone after the last day of shooting’ (1st AD A).

Role ambiguity is reflected in the 1st AD’s uncertainty about their authority during the shoot. This starts with the fact that 1st ADs, even though they need to manage the shoot, cannot hire, evaluate or fire anyone who is working on the shoot, except for their own assistant(s). As one of the respondents expresses it: ‘As a 1st AD you are the store manager, but you did not hire the personnel’ (1st AD M). In addition, the 1st AD is said to be the manager on the set even though they do not have the formal authority to make decisions that have direct financial or creative consequences. As a result, 1st ADs often have to take charge without knowing whether they actually have the authority to do so. Although it is ambiguous whether certain decision-making actions belong to their role, they do assume this responsibility in order to achieve their overall objective of finishing the shoot on time and within budget. This is illustrated by the following quote:

Producers and director often say: ‘during the shoot he is the boss’. This does not mean that I am in charge of the money, nor does it mean that I am in charge of the content because I really am not. However, you do intervene because you have to. You are involved in the money indirectly and the content indirectly and make a contribution. (1st AD H)

When the boundaries between roles are not clear at the start of the project, role ambiguity creates a situation in which 1st ADs run the risk of interpreting, and consequently enacting, their role either too narrowly or too broadly. In the former, they are criticized for not taking enough responsibility, whereas in the latter, they are criticized for meddling in the affairs of the producer and/or director. The following quote illustrates how role ambiguity, in the sense of have a too narrow interpretation of the 1st AD role in relation to that of the director, can have negative consequences:

Last year I was dismissed from a project because it was not going very well. The days [as planned in the shooting schedule] were not realistic and the director felt he had to do my job. I did not think so, but that was a difference in opinion. If there is a conflict between these two individuals, the 1st AD is the first to go. (1st AD K)

### The 1st assistant director and role definition

The sections above show that dual-leadership structures, and their accompanying multiple reporting lines, create role conflict and role ambiguity for the 1st AD. Yet role ambiguity and role conflict also created the need and/or space for 1st ADs to define their role. Nearly all 1st ADs we interviewed provided statements that refer to situations in which they engaged in role definition. Note that in the previous sections a number of quotes about role conflict and role ambiguity already included references to role definition behavior by 1st AD. When talking about role ambiguity (and to a lesser extend role conflict), 1st ADs often also discussed how they dealt with this by engaging in role definition behavior. In this section, we further build on this by focusing on the positive effects of role conflict and role ambiguity on managing conflicts between the dual leaders, resulting from the space they provide for 1st ADs to expansively define their role.

First of all, the degree to which 1st ADs define their role partially depends on their experience. For example, sometimes a producer makes the planning of the shoot in the pre-production phase without closely involving the 1st AD. The more experienced 1st ADs make sure that they are actively involved in making the planning already in the pre-production phase. In addition, because of their seniority in the industry they are more likely to speak up when they believe the planning of the shoot is inefficient or unrealistic. This is illustrated by the following quote: ‘You need to give it [the planning] your own twist. For the films in which I was involved, I have always had my say in the planning, but I can imagine that others don’t dare to insist … you need to have been around in the industry for a while’ (1st AD C). In any case, whenever they define their role, 1st ADs need to ensure that they act, or at least appear to be acting, in the interest of both the producer and the director:

When he [the director] feels the producer breathing down his neck, I will try to bring relief where possible. By solving problems, providing solutions that are acceptable to both. At that moment, you are actually mediating. It’s not that I will act against the producer, but it is more like I will help the director to search for a solution or provide him with arguments with which he can go to the producer. (1st AD L)

Although some 1st ADs have an inclination to side with either the producer or the director, the best ones act as a neutral force that occupies the space between them. According to one of the directors: ‘It’s a bit of a crossover role between direction and production. You have 1st ADs that side mostly with production, but you also have those that side mostly with directing. The best ones are those that operate autonomously in between’ (Director N). The opportunities 1st ADs have for defining their role depends on the space they receive from one or both of the dual leaders. On the one hand, the more producers and/or directors enact their role narrowly, the more room for maneuver they leave for 1st ADs to balance their conflicting interests. On the other hand, the more producers and/or directors define their role expansively, possibly in a forceful attempt to resolve conflicting interests, the less space there is for 1st ADs to define their role in the direction of the dual leaders’ task territories:

That’s what happened to me recently, a director wanted to shoot one last shot at the [airport] terminal … He really wanted that last shot, and at that moment he overruled the production department. That was a bit of a hostile atmosphere. The director said: ‘I’m responsible for this film, my name is on it, so I want that shot.’ The producer wanted to finish within schedule, but eventually the director got his way, because we did do that shot, but the production department was not very happy. I think the director overruled the production manager. (1st AD K)

In situations in which the director and producer regularly cross the boundary between the domains of art and commerce, the 1st AD runs the risk of seeing his or her own role crushed between them, and reduced to running errands. At that point, 1st ADs need to step up and actively intervene by defining their role expansively. However, this is not without risk because by doing so they might – inadvertently or not – invade the role space of either or both the dual leaders:

When it affects the film, I have a direct mediating role between the producer and director. In those cases, I call the shots, and say what we need to do: ‘Shut up and shoot.’ Sometimes you tell the producer: ‘Now you have to be quiet and leave, because if you miss this shot it will be bad for the film.’ In most cases, they will come to you afterwards to say that you were right, and add that they had to say something because it is their role to do so. They have to show who they are in relation to the director. (1st AD F)

An important task of 1st ADs is to make sure that producers and directors continue on good terms when the film shoot runs into problems, as they often inevitably do. In some cases, when directors and producers do not communicate directly, 1st ADs can avoid a dual-leadership conflict by strategically providing or withholding information. In most cases, 1st ADs act as the harbinger of bad news to each of the dual leaders, so producers and directors do not have to accuse each other directly when things run awry. This is illustrated by the following 1st AD:

Because I am the transmitter of bad news, directors and line producers can discuss things in a normal way … The bad news has already landed, which allows them to proceed in a normal way. This is important since they are the ones that eventually have to come to an agreement. (1st AD D)

In conclusion, 1st ADs suffer from role conflict and role ambiguity as a result of being positioned hierarchically below and between dual leaders, and having to report to and being accountable to both. At the same time, this position provides them with opportunities to engage in expansive role definition to alleviate dual-leadership conflicts that might split the organization in two along the art versus commerce divide. In the next section, these findings and their implications are discussed.

## Conclusion and discussion

In this article, we studied how role conflict and role ambiguity impacts role definition by individuals occupying a position between and hierarchically below two superiors in PBOs with a dual-leadership structure. Our empirical study focused on the role of 1st ADs in the film industry. The responsibility of 1st ADs is to execute the planning of the film shoot, and coordinate the activities of the different departments on the film set. The 1st AD occupies a position between and hierarchically below the director and producer. The director and producer form the dual leadership in film projects, and are the main representatives of the artistic and commercial objectives of the organization, respectively, which are often hard to reconcile.

As a result of their particular position, 1st ADs are likely to suffer from role conflict and role ambiguity. First, they can experience role conflict because the expectations projected on them by the producer may be inconsistent, or even diametrically opposed, to those of the director. Second, they are likely to face role ambiguity because the boundaries of the 1st AD role, especially in relation to that of the producer and director, are unclear. However, role conflict and role ambiguity also provide 1st ADs with opportunities to define their role expansively. By doing so, 1st ADs can increase their ability to act as a buffer between the dual leaders. It should be emphasized that, as opposed to stepping in as a mediator in an ad hoc manner once conflicts between dual leaders arise, 1st ADs occupy a structural role as a buffer between dual leaders throughout the project. Moreover, the more the director and producer employ a narrow definition of their respective roles, the more space they leave for the 1st AD, and the more likely it is that positive effects ensue from the 1st AD facilitating collaboration between the dual leaders, and bridging the artistic and commercial sides of the organization.

Our study has three main theoretical contributions. First, we contribute to role theory by showing that role conflict and role ambiguity do not exclusively have negative effects such as stress, lower commitment and lower performance (House and Rizzo, 1972; Jackson and Schuler, 1985; Tubre and Collins, 2000) but can lead to positive effects as well. More specifically, we show that role ambiguity and role conflict can give space for organizational members to define their role expansively, which can benefit the performance of these employees, as well as the wider organization. Role conflict and role ambiguity are conceptually more closely linked to task conflict than relationship conflict (Jehn, 1995). Jehn (1995) suggests that, although relationship conflict is likely to be detrimental to organizational performance, task conflict might actually be beneficial. However, empirical studies have produced mixed evidence of the validity of this thesis (see, for instance, De Dreu and Weingart, 2003). Our study provides specific support for Jehn’s thesis in the context of organizations with dual-leadership structures by showing how task conflict between executives in dual-leadership structures, through creating role conflict and role ambiguity at lower hierarchical levels of the organization, can have positive effects.

Second, we contribute to the dual-leadership literature. Where earlier studies focus mostly on dual leaders, the relationship between them (Reid and Karambayya, 2009), and how role definition by dual leaders affects the organization (Denis et al., 2000), we build on this by studying dual-leadership structure more explicitly in relation to individuals in lower levels of the organizational hierarchy. By doing so, we also respond to an explicit call by Reid and Karambayya (2009) to further investigate how dual-leadership conflicts can disseminate to other levels in the organization. The latter authors focused on how dual leaders can draw in subordinates in lower levels of the hierarchy, or board members in higher levels of the hierarchy, to mediate between them. We build on these ideas by showing how dual leaders, by narrowly defining their roles, can create space for individuals occupying the role hierarchically below and between them to expansively define their role. In turn, this enables them to perform an internal boundary spanning role (Rosenkopf and Nerkar, 2001) that can help alleviate organizational conflicts that can result from having a dual-leadership structure.

Third, our study shows how role definition behavior by organizational members affects other organizational members with interdependent roles. When expansive role definition entails taking over (part of) the role of other organizational members – either vertically or horizontally within the hierarchy – this raises the question of how these other individuals should respond. Our findings suggest that executives in organizations that are characterized by multiple lines of authority could benefit from defining their roles more narrowly, and taking care to avoid invading the jurisdiction of other executives (Bechky, 2003; Lingo and O’Mahony, 2010). Although the interrelatedness of role-crafting behavior by pairs of co-workers in the same organization has received some attention recently (Bakker et al., 2016), our study focuses on the interrelatedness of role-defining by individuals at different levels of the organizational hierarchy.

Our study may also have broader theoretical implications as a result of linking the concepts of plural leadership, role definition and role crafting, especially in ‘pluralistic settings traversed by multiple logics and values, where power relationships are diffuse’ (Denis et al., 2012: 240; see also Denis et al., 2001). The extent to which members of an organization have to deal with multiple lines of authority will increase role stress in the form of role conflict and role ambiguity. If there are multiple lines of authority that are linked to different organizational objectives, this will likely even further increase role stress for those who are involved in the pursuit of multiple objectives at the same time. We suggest that these individuals could respond in two ways.

On the one hand, the members of the organization who are subject to role stress can respond defensively, declining responsibilities or specific demands with the explicit or implicit argument that these are not part of their role. Although such behavior can work well as long as there are no issues or challenges that necessitate cross-organizational collaboration, such behavior will further increase the extent to which the multiple lines of authority will tend to act separately. A self-reinforcing feedback loop could then ensue, with potentially damaging effects to the organization as a whole. The more the multiple leaders act separately, the more likely it will be that particular decisions serve one particular organizational objective, while having a negative effect on achieving others. This will increase the risks of conflict, including interpersonal conflict, which, in turn, makes it more difficult for the leaders to act coherently. Or worse, if there is little trust that the other side will pay enough attention to the objectives of one’s own side, this can lead to each side focusing even more strongly on achieving its own objectives, creating a negative feedback loop, and damaging the performance of the organization as a whole.

On the other hand, our study suggests that there is also a positive response to role stress that can potentially counteract the above-mentioned process, namely expansive role definition by actors who are positioned hierarchically below and between the main representatives of the multiple lines of authority. Our results suggest that this can lead to a positive feedback loop. If the dual leaders allow the individual hierarchically below and between them to define his or her role expansively, this person could act more effectively to reduce the extent to which the multiple lines of authority act separately. This might increase the likelihood that the different organizational objectives are pursued more coherently, leading to a better performance of the organization as a whole. In turn, such positive outcomes will make it more likely that expansive role definition, also by other actors lower in the organizational hierarchy, is more easily accepted and seen less as a threat against which the people at the top need to defend themselves.

### Implications for practice

Our study has a number of practical implications, the first of which is to suggest that organizations could profit from designing structures in such a way that it leaves space for lower-level organizational members, who can mediate between dual or multiple leaders by expanding their role, while taking into account how leaders can directly and indirectly facilitate such role defining or crafting. Second, in a more general sense, because employees, also in non-project-based organizations, tend to have more ‘boundaryless careers’ (Arthur and Rousseau, 1996) and increasingly less stable, defined and demarcated roles, they have to be able to, or learn how to, proactively define or craft their own roles. Managers, in turn, need to learn how to accommodate the positive aspects of expansive role definition and crafting while at the same time mitigating its potentially negative effects on organizational stability, especially as a result of discontent by those organizational members whose roles are being invaded.

### Boundary conditions, limitations and suggestions for future research

There are a number of boundary conditions with respect to the conclusions of our study. First, it should be noted that role ambiguity and role conflict is not always beneficial, or that expansive role definition as a response is always a good thing. Our results show that in a particular organizational context, and given particular responses by actors in adjacent roles, expansive role definition can benefit the organization. Second, it seems likely that the positive effects of expansive role definition or role crafting on organizational processes will not occur if role conflict or role ambiguity rises to extreme levels. If they are too high, the disadvantages for the efficiency of normal organizational processes are likely to outweigh the advantages. Third, if the representatives of each line of authority completely isolate themselves from what goes on in the other half of the organization, this will likely reduce the scope and effectiveness of the actor who defines her/his role expansively and have a negative effect on organizational performance. Finally, we looked at individuals hierarchically immediately below and in between the dual leaders. Such an individual does not need to occupy a role exactly in the middle, but if the role does not include at least some responsibilities in relation to each of the dual leaders, it would be much harder to define the role in the way described in this article.

This study has a number of limitations that also suggest directions for future research. First, because we exclusively focused on the Dutch film industry, caution may need to be exercised in generalizing these findings to other international contexts or different industries. For example, in Italy directors are more powerful than producers, whereas in Hollywood one finds the opposite (Delmestri et al., 2005). Even though the balance of power between the director and producer might vary across countries, the roles and organizational structures of film productions are rather similar. We therefore expect that our findings are largely generalizable to film industries in other countries. Moreover, PBOs in other industries, such as construction, can have characteristics that are different from the ones described in this study. As mentioned before, the individuals in these organizations are likely to be less emotionally invested than is the case in the film industry (Rowlands and Handy, 2012), which could have the effect of dampening conflicts, or at least making it less likely that negative feedback loops run out of control.

Second, this study focused on a particular organizational structure, namely dual leadership. Besides dual-leadership structures, there are many other organizational structures in which individuals have to deal with multiple reporting lines (Denis et al., 2000; Fjellvaer, 2010; Galbraith, 1974; Sy and D’Annunzio, 2005). Future studies could investigate the extent to which the relationships between role stress, role definition and possibly role crafting, vary with the particular characteristics of the organizational design in which the multiple lines of authority appear. In addition, such studies could also take into account a broader range of roles around the leadership positions. Finally, in the organizations we studied, the dual leaders themselves decided to team up instead of being appointed or selected (Reid and Karambayya, 2009). This implies that, in our context of the film industry, directors and producers trusted each other enough to collaborate. When dual leaders have been brought together by others, the role defining dynamics could play out differently.

Finally, this study focuses on role defining in dual leadership in the context of PBOs. Because temporary PBOs rarely allow role crafting in the sense of more permanently changing task or relational boundaries, further research could explore whether in permanent organizations with multiple lines of authority, role crafting will have similar antecedents and consequences as role definition in the context of our study. Although role definition can be seen as the first phase of role crafting (Sluss et al., 2011), precisely the temporary nature of the organizations we studied made it impossible to determine whether full role crafting had taken place. When the process of role definition had finished, the PBO was at the end of its effective life, and its members moved on to other PBOs. However, evaluating our results in the light of earlier studies on role crafting (Sluss et al., 2011; Wrzesniewski and Dutton, 2001) suggests that the processes of role definition in PBOs are not that different from the processes of role crafting in more permanent organizations.

## Appendix 1: Selected protocol questions

1. You can specify who normally approaches you to join a project?
2. How would you describe your role and associated responsibilities during pre-production, in case you are involved at this stage?
3. How would you describe your role and associated responsibilities during production?
4. To what extent do you know before the start of the project what is expected of you?
5. Who assesses your performance on the set and what criteria are being used?
6. To what extent are these criteria made   explicit before the start of the project?
7. What are the advantages and disadvantages of these criteria being made explicit or not?
8. To whom are you directly accountable?
9. What are your responsibilities towards the director?
10. To what extent are there conflicts on the set between 1st ADs and directors?
11. Can you describe how these conflicts are resolved?
12. What are your responsibilities towards the producer?
13. To what extent are there conflicts on the set between 1st ADs and producers?
14. Can you describe how these conflicts are resolved?
15. How would you describe the relationship between producer and director?
16. Which bottlenecks did you experience between the interests of the director and the interests of the producer?
17. Can you give any examples of situations in which your responsibilities to the producer were difficult to reconcile with your responsibilities to the director?
18. How did you deal with these situations?
19. Can you give any examples of a situation where you had to mediate between the producer and the director?
20. What is the impact of conflicts on the set on the performance of the project organization as a whole?
  Notes: AD = assistant director.
